# A simulation study: Improved ratio-in-regression type variance estimator based on dual use of auxiliary variable under simple random sampling

**DOI:** 10.1371/journal.pone.0276540

**Published:** 2022-11-18

**Authors:** Sohaib Ahmad, Sardar Hussain, Kalim Ullah, Erum Zahid, Muhammad Aamir, Javid Shabbir, Zubair Ahmad, Huda M. Alshanbari, Wejdan Alajlan

**Affiliations:** 1 Department of Statistics, Abdul Wali Khan University, Mardan, Pakistan; 2 Department of Statistics, Quaid-i-Azam University Islamabad, Pakistan; 3 Foundation University Medical College, Foundation University School of Health Sciences, DHA-I, Islamabad, Pakistan; 4 Department of Applied Mathematics and Statistics, Institute of Space Techonalogy, Islamabad, Pakistan; 5 Department of Statistics, University of Wah at Wah Cantt, Wah Cantt, Pakistan; 6 Department of Mathematical Sciences, College of Science, Princess Nourah bint Abdulrahman University, Riyadh, Saudi Arabia; Amity University - Lucknow Campus, INDIA

## Abstract

In this article, we proposed an improved finite population variance estimator based on simple random sampling using dual auxiliary information. Mathematical expressions of the proposed and existing estimators are obtained up to the first order of approximation. Two real data sets are used to examine the performances of a new improved proposed estimator. A simulation study is also recognized to assess the robustness and generalizability of the proposed estimator. From the result of real data sets and simulation study, it is examining that the proposed estimator give minimum mean square error and percentage relative efficiency are higher than all existing counterparts, which shown the importance of new improved estimator. The theoretical and numerical result illustrated that the proposed variance estimator based on simple random sampling using dual auxiliary information has the best among all existing estimators.

## 1. Introduction

In survey sampling, it is well known fact that efficiency of population parameters estimates can be improved by the use of auxiliary information, providing it is highly associated with the study variable. Auxiliary information plays a vital role in parameter selection and estimation to achieve efficient estimates of the unknown population parameters. Many authors have suggested estimators based on original auxiliary information. In fact, appropriate use of auxiliary variable in survey sampling results in significant decrease in the variance of the estimator of unknown population parameter(s). Variance is one of the features that can be used to describe a statistical population. Brilliant efforts have been taken to estimate this characteristic as accurately as possible. The goal is to reduce the scattering of the calculated estimate when it is obtained from many available samples. Furthermore, the importance of using information from one or more auxiliary variables is to improve estimator efficiency. Many attempts have previously been made in this direction, including use of the ratio and transformed ratio-product-type. By utilizing the association among the study variable and auxiliary variable, the survey statisticians have shaped effective ratio and product estimators for population variance. The estimation of population variance of the study variable is required in a variety of scenarios in application. In a variety of fields, including agriculture, medicine, biology, and business, where we encounter populations that are likely to be skewed, variance estimate for the population is important.

Variations can be found in our daily lives throughout the world. It is a common belief that no two things or people are exactly the same. For example, a farmer must have a good alertness of how climate geographies change over time to time in order to determine how to plant his lot. For proper treatment, a doctor must have a detailed realization of differences in human hypertension and fever. Many authors suggested different types of estimators for variance including Das [1], Prasad and Singh [2], Swain and Mishra [3], Ahmad and Shabbir [4], Kadilar and Cingi [5], Subramani and Kumarapandiyan [6], Singh and Solanki [7], Yadav et al. [8], Singh et al. [9], Yadav and Kadilar [10], Adichwal et al. [11,12], Yasmeen et al. [13], Zaman [14,15], Zaman and Bulut [16], Zaman et al. [17,18], Bhushan et al. [19], Shahzad et al. [20], Ahmad et al. [21], Ozturk [22], Ozturk and Demirel [23], Frey and Feeman [24], Zamanzade and Vock [25], Mahdizadeh and Zamanzade [26,27], Bhushan et al. [28], Bhushan et al. [29], Zaman and Bulut [30,31], Yadav and Zaman [32], Zaman and Kadilar [33], Zaman and Dunder [34] and Singh and Khalid [35]. We requisite to estimate population parameters for numerous variables in multidisciplinary surveys, such as house lengths, income level, and the number of unemployed people, in socio-economic surveys. The goal of this study is to offer a strategy for estimating population variance using a dual auxiliary variable in simple random sampling. We found no contribution on variance estimation using dual auxiliary variables under simple random sampling in the literature.

## 2. Notations and symbols

Consider *ψ* = (*ψ*_1_, *ψ*_2_,…,*ψ*_*N*_) be a population comprise of size *N* elements, let a sample of size *n* is chosen from *ψ* by using simple random sampling without replacement (SRSWOR). Let *Y*, *X* and *R*_*x*_ represent the study variable, auxiliary variable and rank of the auxiliary variable respectively. Consider population variance of the study variable *Y*, auxiliary variable *X* and rank of the auxiliary variable *R*_*x*_ is denoted by $S_{y}^{2},S_{x}^{2}$ and $S_{rx}^{2}$. Let *y*_*i*_, *x*_*i*_ and *r*_*xi*_, be the sample for the *i*^*th*^ units correspondingly. Let $s_{y}^{2}=\frac{{\sum_{i=1}^{n}\left(y_{i}-\bar{y}\right)}^{2}}{n-1},s_{x}^{2}=\frac{{\sum_{i=1}^{n}\left(x_{i}-\bar{x}\right)}^{2}}{n-1},s_{rx}^{2}=\frac{{\sum_{i=1}^{n}\left({rx}_{i}-{\bar{r}}_{x}\right)}^{2}}{n-1}$, be the sample variances. $S_{y}^{2}=\frac{{\sum_{i=1}^{N}\left(y_{i}-\bar{Y}\right)}^{2}}{N-1},S_{x}^{2}=\frac{{\sum_{i=1}^{N}\left(x_{i}-\bar{X}\right)}^{2}}{N-1},S_{rx}^{2}=\frac{{\sum_{i=1}^{N}\left(r_{xi}-{\bar{R}}_{x}\right)}^{2}}{N-1}$, of *Y*, *X* and *R*_*x*_ be the corresponding variances. To derive the properties of the suggested estimator, we have the following error terms.


$$Let\;\xi_{o}=\frac{s_{y}^{2}-S_{y}^{2}}{S_{y}^{2}},\xi_{1}=\frac{s_{x}^{2}-S_{x}^{2}}{S_{x}^{2}},\xi_{2}=\frac{s_{rx}^{2}-S_{rx}^{2}}{S_{rx}^{2}},\;E(\xi_{i})=0,\;for\;(i=0,1,2).$$

$$E(\xi_{o}^{2})=ʎ{ƛ}_{400}^{*},E(\xi_{1}^{2})=ʎ{ƛ}_{040}^{*},E(\xi_{2}^{2})=ʎ{ƛ}_{004}^{*},E(\xi_{o}\xi_{1})=ʎ{ƛ}_{220}^{*},E(\xi_{0}\xi_{2})=ʎ{ƛ}_{202}^{*},E(\xi_{1}\xi_{2})=ʎ{ƛ}_{022,}^{*}$$

$${ƛ}_{rst}^{*}=({ƛ}_{rst}-1),\;where\;ʎ=\left(\frac{1}{n}-\frac{1}{N}\right).$$

$${ƛ}_{rst}=\frac{\upsilon_{rst}}{\upsilon_{200}^{\frac{r}{2}}\upsilon_{020}^{\frac{s}{2}}\upsilon_{002}^{\frac{t}{2}}},\upsilon_{rst}=\frac{\sum ({y_{hi}-{\bar{Y}}_{h})}^{s}({x_{hi}-{\bar{X}}_{h})}^{s}{\left(r_{x_{hi}}-{\bar{R}}_{xh}\right)}^{t}}{N-1}.$$

Where r, s and *t* be the positive number and υ_200_, υ_020_, υ_002_ are the second order moments about means of *Y*, *X* and rank of *X*, and *ƛ*_*rst*_ is the moment ratio.

## 3. Existing estimators

In this section, we discussed several well-known estimators of population variance.

- The usual estimator, is given by:
$${\hat{\bar{Y}}}_{0}=\frac{{\sum_{i=1}^{n}\left(y_{i}-\bar{Y}\right)}^{2}}{n-1}$$ (1)
  The variance of ${\hat{\bar{Y}}}_{0}$, is given by:
$$Var({\hat{\bar{Y}}}_{0})=ʎS_{y}^{4}{ƛ}_{400}^{*}$$ (2)
- Isaki [36] suggested ratio estimator, is given by:
$${\hat{\bar{Y}}}_{1}=s_{y}^{2}\left(\frac{S_{x}^{2}}{s_{x}^{2}}\right)$$ (3)
  The properties of ${\hat{\bar{Y}}}_{1}$, is given as:
$$Bias({\hat{\bar{Y}}}_{1})=ʎS_{y}^{2}\{{ƛ}_{040}^{*}-{ƛ}_{220}^{*}\},$$
  and
$$MSE({\hat{\bar{Y}}}_{1})≅ʎS_{y}^{4}\{{ƛ}_{400}^{*}+{ƛ}_{040}^{*}-2{ƛ}_{220}^{*}\}.$$ (4)
- The difference estimator, is given by:
$${\hat{\bar{Y}}}_{2}=s_{y}^{2}+\Psi_{11}(S_{x}^{2}-s_{x}^{2}).$$ (5)
  Where
$$\Psi_{11}=[\frac{S_{y}^{2}{ƛ}_{220}^{*}}{S_{x}^{2}{ƛ}_{040}^{*}}].$$
  The minimum variance at the optimal value of *Ψ*_11_, is given by:
$$Var({\hat{\bar{Y}}}_{2})={ʎS}_{y}^{4}{ƛ}_{400}^{*}(1-\rho^{2}).$$ (6)
  Where
$$\rho =\frac{{ƛ}_{220}^{*}}{\sqrt{{ƛ}_{400}^{*}}\sqrt{{ƛ}_{040}^{*}}}.$$
- Rao [37] difference type estimator, is given by:
$${\hat{\bar{Y}}}_{3}=\{{Ϣ}_{1}s_{y}^{2}+{Ϣ}_{2}(S_{x}^{2}-s_{x}^{2})\},$$ (7)
  where
$${Ϣ}_{1}=\frac{{ƛ}_{040}^{*}}{ʎ\{{ƛ}_{400}^{*}{ƛ}_{040}^{*}-{ƛ}_{220}^{*2}\}+{ƛ}_{040}^{*}},$$
$${Ϣ}_{2}=\frac{S_{y}^{2}{ƛ}_{220}^{*}}{S_{x}^{2}\{ʎ({ƛ}_{400}^{*}{ƛ}_{040}^{*}-{ƛ}_{220}^{*2})+{ƛ}_{040}^{*}\}},$$
  The properties at the optimal value of Ϣ_1_ and Ϣ_2_, are given by:
$$Bias({\hat{\bar{Y}}}_{3})=S_{y}^{2}\{\frac{{ƛ}_{040}^{*}}{ʎ\{{ƛ}_{400}^{*}{ƛ}_{040}^{*}-{ƛ}_{220}^{*2})+{ƛ}_{040}^{*}}-1\},$$
  and
$$MSE\;({\hat{\bar{Y}}}_{3})=\frac{ʎS_{y}^{4}[{ƛ}_{400}^{*}{ƛ}_{040}^{*}-{ƛ}_{220}^{*2}]}{ʎ[{ƛ}_{400}^{*}{ƛ}_{040}^{*}-{ƛ}_{220}^{*2}]+{ƛ}_{040}^{*}}$$ (8)
- Singh et al. [38] suggested the following estimators:
$${\hat{\bar{Y}}}_{4}=S_{y}^{2}exp\left\{\frac{S_{x}^{2}-s_{x}^{2}}{S_{x}^{2}+s_{x}^{2}}\right\}$$ (9)
  The properties of ${\hat{\bar{Y}}}_{4}$, are given by:
$$Bias({\hat{\bar{Y}}}_{4})=ʎS_{y}^{2}\{\frac{3}{8}{ƛ}_{040}^{*}-\frac{1}{2}{ƛ}_{220}^{*}\},$$ (10)
$$MSE\;({\hat{\bar{Y}}}_{4})=ʎS_{y}^{4}\left[{ƛ}_{400}^{*}+\frac{1}{4}{ƛ}_{040}^{*}-{ƛ}_{220}^{*}\right]$$ (11)
- Grover and Kaur [39], suggested the following estimator, is given by:
$${\hat{\bar{Y}}}_{5}=[{Ϣ}_{3}s_{y}^{2}+{Ϣ}_{4}(S_{x}^{2}-s_{x}^{2})]exp\left(\frac{S_{x}^{2}-s_{x}^{2}}{S_{x}^{2}+s_{x}^{2}}\right)$$ (12)
$$Bias\left({\hat{\bar{Y}}}_{5}\right)=[-S_{y}^{2}+{Ϣ}_{3}S_{y}^{2}\left\{1+\frac{3}{8}{ƛ}_{040}^{*}-\frac{1}{2}{ƛ}_{220}^{*}\right\}+\frac{1}{2}{Ϣ}_{4}S_{x}^{2}ʎ{ƛ}_{040}^{*}],$$
  Where Ϣ_3_ and Ϣ_4_ are the unknown constant
$${Ϣ}_{3}=\frac{\left\{8{ƛ}_{040}^{*}-ʎ{ƛ}_{040}^{*2}\right\}}{8[ʎ({ƛ}_{400}^{*}{ƛ}_{040}^{*}-{ƛ}_{220}^{*2})+{ƛ}_{040}^{*}]},$$
$${Ϣ}_{4}=\frac{S_{y}^{2}(ʎ{ƛ}_{040}^{*2}-ʎ{ƛ}_{040}^{*}{ƛ}_{220}^{*}+8{ƛ}_{220}^{*}-4\{{ƛ}_{040}^{*}-ʎ({ƛ}_{400}^{*}{ƛ}_{040}^{*}-{ƛ}_{220}^{*2})\})}{8S_{x}^{2}[ʎ\{{ƛ}_{400}^{*}{ƛ}_{040}^{*}-{ƛ}_{220}^{*2}\}+{ƛ}_{040}^{*}]}.$$
  The minimum MSE at the optimum values of Ϣ_3_, Ϣ_4_, are given by:
$$MSE({\hat{\bar{Y}}}_{5})=\frac{{ʎS}_{y}^{2}[64\{{ƛ}_{400}^{*}{ƛ}_{040}^{*}-{ƛ}_{220}^{*2}\}-ʎ{ƛ}_{040}^{*3}-16ʎ{ƛ}_{040}^{*}\{{ƛ}_{400}^{*}{ƛ}_{040}^{*}-{ƛ}_{220}^{*2}\}]}{64\{ʎ({ƛ}_{400}^{*}{ƛ}_{040}^{*}-{ƛ}_{220}^{*2})+{ƛ}_{040}^{*}\}}$$ (13)
- Ahmad et al. [40], suggested the following estimator, given by:
$${\hat{\bar{Y}}}_{6}=[{Ϣ}_{5}s_{y}^{2}+{Ϣ}_{6}(S_{x}^{2}-s_{x}^{2})+{Ϣ}_{7}(S_{rx}^{2}-s_{rx}^{2})]exp\left(\frac{S_{x}^{2}-s_{x}^{2}}{S_{x}^{2}+s_{x}^{2}}\right)$$ (14)
$${\hat{\bar{Y}}}_{6}=[{Ϣ}_{5}s_{y}^{2}(1+\xi_{o})-{Ϣ}_{6}\xi_{1}-{Ϣ}_{7}\xi_{2}](1-\frac{1}{2}{Ϣ}_{6}+\frac{3}{8}{{Ϣ}_{6}}^{2}+\cdots ).$$
  After expanding the above equation, we have
$$({\hat{\bar{Y}}}_{6}-S_{y}^{2})=-S_{y}^{2}+{Ϣ}_{5}S_{y}^{2}+{Ϣ}_{5}S_{y}^{2}\xi_{o}-\frac{1}{2}{Ϣ}_{5}S_{y}^{2}\xi_{1}-{Ϣ}_{6}\xi_{1}-{Ϣ}_{7}\xi_{2}+\frac{3}{8}{Ϣ}_{5}S_{y}^{2}\xi_{1}^{2}+\frac{1}{2}{Ϣ}_{6}\xi_{1}^{2}-\frac{1}{2}{Ϣ}_{5}S_{y}^{2}\xi_{o}\xi_{1}-\frac{1}{2}{Ϣ}_{7}\xi_{1}\xi_{2}$$
  The bias and MSE of ${\hat{\bar{Y}}}_{6}$, are given by:
$$Bias\left({\hat{\bar{Y}}}_{6}\right)≅-S_{y}^{2}+{Ϣ}_{5}S_{y}^{2}\left(1+\frac{3}{8}{ƛ}_{040}^{*}-\frac{1}{2}{ƛ}_{220}^{*}\right)+\frac{1}{2}ʎ({Ϣ}_{6}S_{x}^{2}{ƛ}_{040}^{*}+{Ϣ}_{7}S_{rx}^{2}{ƛ}_{022}^{*}),$$
  The optimal values of Ϣ5, Ϣ6, and Ϣ_7_, are given by:
$${{Ϣ}_{5}}_{\left(optim\right)}=\frac{8-ʎ{ƛ}_{040}^{*}}{{ƛ}_{400}^{*}[8\{\frac{1}{{ƛ}_{400}^{*}}+ʎ({Ɣ}^{*}+1)\}]},$$
$${{Ϣ}_{6}}_{\left(optim\right)}=\frac{S_{y}^{2}[\begin{array}{c} ʎ{ƛ}_{040}^{*}({ƛ}_{040}^{*}{ƛ}_{004}^{*}-{ƛ}_{220}^{*2})+({ƛ}_{004}^{*}{ƛ}_{220}^{*}-{ƛ}_{202}^{*}{ƛ}_{022}^{*})(8-ʎ{ƛ}_{040}^{*})+4{ƛ}_{400}^{*}({ƛ}_{040}^{*}{ƛ}_{004-}^{*}{ƛ}_{022}^{*2})\\ \left\{\left(\frac{-1}{{ƛ}_{400}^{*}})+ʎ({Ɣ}^{*}+1\right)\right\} \end{array}]}{8S_{x}^{2}{ƛ}_{400}^{*}({ƛ}_{040}^{*}{ƛ}_{004}^{*}-{ƛ}_{220}^{*2})\{\left(\frac{1}{{ƛ}_{400}^{*}})+ʎ({Ɣ}^{*}+1\right)\}},$$
  and
$${{Ϣ}_{7}}_{\left(optim\right)}=\frac{S_{y}^{2}[8-ʎ{ƛ}_{040}^{*}]\{{ƛ}_{220}^{*}{ƛ}_{002}^{*}-{ƛ}_{040}^{*}{ƛ}_{202}^{*}\}}{8S_{rx}^{2}{ƛ}_{400}^{*}({ƛ}_{040}^{*}{ƛ}_{004}^{*}-{ƛ}_{220}^{*2})\{\left(\frac{1}{{ƛ}_{400}^{*}})+ʎ({Ɣ}^{*}+1\right)\}}.$$
  The minimum mean square error at the optimal values of ${{Ϣ}_{5}}_{\left(optim\right)},{{Ϣ}_{6}}_{\left(optim\right)}$ and ${{Ϣ}_{7}}_{\left(optim\right)}$, are given by:
$${MSE({\hat{\bar{Y}}}_{6})}_{min}=\frac{ʎS_{y}^{2}[64({Ɣ}^{*}+1)-ʎ(\frac{{ƛ}_{040}^{*2}}{{ƛ}_{400}^{*}})-16ʎ{ƛ}_{040}^{*}({Ɣ}^{*}+1)]}{64\{\frac{1}{{ƛ}_{400}^{*}}+ʎ({Ɣ}^{*}+1)\}},$$ (15)
  where
$${Ɣ}^{*}=\frac{2{ƛ}_{220}^{*}{ƛ}_{202}^{*}{ƛ}_{022}^{*}-{ƛ}_{040}^{*}{ƛ}_{202}^{*2}-{ƛ}_{004}^{*}{ƛ}_{220}^{*2}}{{ƛ}_{400}^{*}({ƛ}_{040}^{*}{ƛ}_{004-}^{*}{ƛ}_{220}^{*2})}$$

## 4. Proposed improved ratio-in-regression type variance estimator

The need for auxiliary variable can boost estimator efficiency during the design and estimation stage. When the association among the study variable and auxiliary variables exist, then rank of the auxiliary variable is also associated with the study variable. Therefore, the rank of the auxiliary variable (that includes rank of the auxiliary variable) can be considered as a novel auxiliary variable, and this evidence may help us in increasing the precision of an estimator. Based on these ideas, we are encouraged to investigate dual use of the auxiliary variable for population variance because it has been examined that there has been no work before. The suggested estimator includes the auxiliary information in the form of an auxiliary variable and in the form of a rank auxiliary variable. There are several estimators for estimating population variance based on original auxiliary variables. The key advantage of our proposed estimator in simple random sampling is that it is more flexible and best than existing estimators. By taking motivation from Ahmad et al. [40] we proposed the following estimator:

$${\hat{\bar{Y}}}_{prop}={Ϣ}_{8}S_{y}^{2}+{Ϣ}_{9}(S_{x}^{2}-s_{x}^{2})\;exp\left(\frac{S_{x}^{2}-s_{x}^{2}}{S_{x}^{2}+s_{x}^{2}}\right)+{Ϣ}_{10}(S_{rx}^{2}-s_{rx}^{2})\;exp\left(\frac{S_{rx}^{2}-s_{rx}^{2}}{S_{rx}^{2}+s_{rx}^{2}}\right)$$ (16)

Simplifying (16), we have

$${\hat{\bar{Y}}}_{prop}={Ϣ}_{8}S_{y}^{2}(1+\xi_{o})-{Ϣ}_{9}S_{x}^{2}\xi_{1}(1-\frac{1}{2}\xi_{1}+\frac{3}{8}\xi_{1}^{2})-{Ϣ}_{10}S_{rx}^{2}\xi_{2}(1-\frac{1}{2}\xi_{2}+\frac{3}{8}\xi_{2}^{2})$$

$${\hat{\bar{Y}}}_{prop}-S_{y}^{2}=({Ϣ}_{8}‐1)S_{y}^{2}+{Ϣ}_{8}S_{y}^{2}\xi_{o}-{Ϣ}_{9}S_{x}^{2}(\xi_{1}-\frac{1}{2}\xi_{1}^{2})-{Ϣ}_{10}S_{rx}^{2}(\xi_{2}-\frac{1}{2}\xi_{2}^{2})$$ (17)

$$Bias({\hat{\bar{Y}}}_{prop})=({Ϣ}_{8}‐1)S_{y}^{2}+\frac{1}{2}{Ϣ}_{9}S_{x}^{2}ʎ{ƛ}_{040}^{*}+\frac{1}{2}{Ϣ}_{10}S_{rx}^{2}ʎ{ƛ}_{004}^{*}$$

Simplify (17), we have

$$MSE({\hat{\bar{Y}}}_{prop})={\left({Ϣ}_{8}-1\right)}^{2}S_{y}^{4}+{Ϣ}_{8}^{2}S_{y}^{4}\xi_{0}^{2}+{Ϣ}_{9}^{2}S_{x}^{4}\xi_{1}^{2}+{Ϣ}_{10}^{2}S_{rx}^{4}\xi_{2}^{2}+2\;{Ϣ}_{8}({Ϣ}_{8}‐1)S_{y}^{4}\xi_{o}‐2\;({Ϣ}_{8}‐1){Ϣ}_{9}S_{y}^{2}S_{x}^{2}(\xi_{1}-\frac{1}{2}\xi_{1}^{2})–2({Ϣ}_{8}‐1){Ϣ}_{10}S_{y}^{2}S_{rx}^{2}(\xi_{2}-\frac{1}{2}\xi_{2}^{2})‐2\;{Ϣ}_{8}{Ϣ}_{9}S_{y}^{2}S_{x}^{2}(\xi_{0}\xi_{1})-2\;{Ϣ}_{8}{Ϣ}_{10}S_{y}^{2}S_{rx}^{2}(\xi_{0}\xi_{2})+2\;{Ϣ}_{9}{Ϣ}_{10}S_{y}^{2}S_{rx}^{2}(\xi_{1}\xi_{2})$$

$$MSE({\hat{\bar{Y}}}_{prop})={\left({Ϣ}_{8}-1\right)}^{2}S_{y}^{4}+{Ϣ}_{8}^{2}S_{y}^{4}ʎ{ƛ}_{400}^{*}+{Ϣ}_{9}^{2}S_{x}^{4}{ʎƛ}_{040}^{*}+{Ϣ}_{10}^{2}S_{rx}^{4}{ʎƛ}_{004}^{*}+2({Ϣ}_{8}{Ϣ}_{9}-{Ϣ}_{10})S_{y}^{2}S_{x}^{2}\frac{{ʎƛ}_{040}^{*}}{2}+2({Ϣ}_{8}{Ϣ}_{10}-{Ϣ}_{10})S_{y}^{2}S_{rx}^{2}\frac{{ʎƛ}_{004}^{*}}{2}‐2{Ϣ}_{8}{Ϣ}_{9}S_{y}^{2}S_{x}^{2}ʎ{ƛ}_{220}^{*}‐2{Ϣ}_{8}{Ϣ}_{10}S_{y}^{2}S_{rx}^{2}ʎ{ƛ}_{202}^{*}+2{Ϣ}_{9}{Ϣ}_{10}S_{y}^{2}S_{rx}^{4}ʎ{ƛ}_{022}^{*}$$ (18)

The optimum values of Ϣ_8_, Ϣ_9_ and Ϣ_10_ are given by:

Ϣ8=ʎ[Ɲ1ƛ400*−2(ƴ11+Ɲ11)]−4ƛ400*ʎ(4{(Ɣ*+1)+(ƴ11+Ɲ11)}−Ɲ11ƛ400*)+4ƛ400*,

Ϣ9=Sy2(2ʎƛ400*ƛ004*(ƛ040*−ƛ022*)+ʎƛ040*ƛ202*(ƛ004*−2ƛ202*)+ʎƛ004*ƛ220*(−ƛ004*+2ƛ202*)+4(ƛ004*ƛ220*−ƛ202*ƛ022*))Sx2ƛ400*(ƛ040*ƛ004*−ƛ022*2){ʎ(4{(Ɣ*+1)+(ƴ11+Ɲ11))−Ɲ1ƛ400*)+4ƛ400*},

Ϣ10=Sy2(2ʎƛ400*ƛ040*(ƛ004*−ƛ022*)+ʎƛ040*ƛ202*(−ƛ040*+2ƛ220*)+ʎƛ004*ƛ220*(ƛ040*−2ƛ220*)+4(ƛ040*ƛ202*−ƛ220*ƛ022*))Srx2ƛ400*(ƛ040*ƛ004*−ƛ022*2){ʎ(4{(Ɣ*+1)+(ƴ11+Ɲ11)}−Ɲ1ƛ400*)+Ɲ1ƛ400*}.

Where,

$${ƴ}_{1}=\frac{{ƛ}_{202}^{*2}{ƛ}_{040}^{*2}+{ƛ}_{004}^{*2}{ƛ}_{220}^{*2}-2{ƛ}_{040}^{*}{ƛ}_{004}^{*}{ƛ}_{220}^{*}{ƛ}_{202}^{*}}{{ƛ}_{400({ƛ}_{040}^{*}{ƛ}_{004}^{*}-{ƛ}_{022}^{*2})}^{*}},{Ɲ}_{1}=\frac{{ƛ}_{040}^{*}{ƛ}_{004}^{*}({ƛ}_{040}^{*}+{ƛ}_{004}^{*}-2{ƛ}_{022}^{*})}{{ƛ}_{040}^{*}{ƛ}_{004}^{*}-{ƛ}_{022}^{*2}},$$

$${ƴ}_{11}=\frac{{ƛ}_{004}^{*}{ƛ}_{220}^{*}({ƛ}_{040}^{*}-{ƛ}_{022}^{*})}{{ƛ}_{400}^{*}({ƛ}_{040}^{*}{ƛ}_{004}^{*}-{ƛ}_{022}^{*2})},{Ɲ}_{11}=\frac{{ƛ}_{040}^{*}{ƛ}_{202}^{*}({ƛ}_{004}^{*}-{ƛ}_{022}^{*})}{{ƛ}_{400}^{*}({ƛ}_{040}^{*}{ƛ}_{004}^{*}-{ƛ}_{022}^{*2})},{Ɣ}^{*}=\frac{2{ƛ}_{220}^{*}{ƛ}_{202}^{*}{ƛ}_{022}^{*}-{ƛ}_{040}^{*}{ƛ}_{202}^{*2}-{ƛ}_{004}^{*}{ƛ}_{220}^{*2}}{{ƛ}_{400}^{*}({ƛ}_{040}^{*}{ƛ}_{004-}^{*}{ƛ}_{022}^{*2})}.$$

The minimum MSE at the optimum values of Ϣ_8_, Ϣ_9_ and Ϣ_10_ in (18), we have:

MSE(Y¯^prop)min=ʎSy4[(ƴ1−Ɲ1)ʎ+(Ɣ*+1)]ʎ[4{(Ɣ*+1)+(ƴ11+Ɲ11)}−Ɲ1ƛ400*]+4ƛ400* (19)

## 5. Efficiency comparison

In this section, the theoretical comparison of the suggested estimator with existing estimators is considered:

- From (2) and (19),
$$MSE({\hat{\bar{Y}}}_{prop})<Var({\hat{\bar{Y}}}_{0})\;\mathrm{if}$$
$$Var({\hat{\bar{Y}}}_{0})-MSE({\hat{\bar{Y}}}_{prop})>0$$
$$ʎS_{y}^{4}({ƛ}_{400}^{*}-Ɱ)>0$$
  where
Ɱ=[(ƴ1−Ɲ1)ʎ+(Ɣ*+1)]ʎ[4{(Ɣ*+1)+(ƴ11+Ɲ11)}−Ɲ1ƛ400*]+4ƛ400*
- From (4) and (19),
$$MSE({\hat{\bar{Y}}}_{prop})<MSE({\hat{\bar{Y}}}_{1})\;\mathrm{if}$$
$$MSE({\hat{\bar{Y}}}_{1})-MSE({\hat{\bar{Y}}}_{prop})>0$$
$$ʎS_{y}^{4}(\{{ƛ}_{400}^{*}+{ƛ}_{040}^{*}-2{ƛ}_{220}^{*}\}-Ɱ)>0$$
- From (6) and (19),
$$MSE({\hat{\bar{Y}}}_{prop})<Var({\hat{\bar{Y}}}_{2})\;if$$
$$Var({\hat{\bar{Y}}}_{2})-MSE({\hat{\bar{Y}}}_{prop})>0$$
$$ʎS_{y}^{4}({ƛ}_{400}^{*}(1-\rho^{2})-Ɱ)>0$$
- From (8) and (19),
$$MSE({\hat{\bar{Y}}}_{prop})<MSE({\hat{\bar{Y}}}_{3})\;if$$
$$MSE({\hat{\bar{Y}}}_{3})-MSE({\hat{\bar{Y}}}_{prop})>0$$
$$ʎS_{y}^{4}\left(\frac{\left[{ƛ}_{400}^{*}{ƛ}_{040}^{*}-{ƛ}_{220}^{*2}\right]}{ʎ[{ƛ}_{400}^{*}{ƛ}_{040}^{*}-{ƛ}_{220}^{*2}]+{ƛ}_{040}^{*}}-Ɱ\right)>0$$
- From (11) and (19),
$$MSE({\hat{\bar{Y}}}_{prop})<MSE({\hat{\bar{Y}}}_{4})\;\mathrm{if}$$
$$MSE({\hat{\bar{Y}}}_{4})-MSE({\hat{\bar{Y}}}_{prop})>0$$
$$ʎS_{y}^{4}\left(\{{ƛ}_{400}^{*}+\frac{1}{4}{ƛ}_{040}^{*}-{ƛ}_{220}^{*}\}-Ɱ\right)>0$$
- From (13) and (19),
$$MSE({\hat{\bar{Y}}}_{prop})<MSE({\hat{\bar{Y}}}_{5})\;\mathrm{if}$$
$$MSE({\hat{\bar{Y}}}_{5})-MSE({\hat{\bar{Y}}}_{prop})>0$$
$$ʎS_{y}^{4}\left(\frac{64\{{ƛ}_{400}^{*}{ƛ}_{040}^{*}-{ƛ}_{220}^{*2}\}-ʎ{ƛ}_{040}^{*3}-16ʎ{ƛ}_{040}^{*}\{{ƛ}_{400}^{*}{ƛ}_{040}^{*}-{ƛ}_{220}^{*2}\}}{64\{ʎ({ƛ}_{400}^{*}{ƛ}_{040}^{*}-{ƛ}_{220}^{*2})+{ƛ}_{040}^{*}\}}-Ɱ\right)>0$$
- From (15) and (19),
$$MSE({\hat{\bar{Y}}}_{prop})<MSE({\hat{\bar{Y}}}_{6})\;\mathrm{if}$$
$$MSE({\hat{\bar{Y}}}_{6})-MSE({\hat{\bar{Y}}}_{prop})>0$$
$$ʎS_{y}^{4}\left(\frac{64({Ɣ}^{*}+1)-ʎ(\frac{{ƛ}_{040}^{*2}}{{ƛ}_{400}^{*}})-16\;ʎ{ƛ}_{040}^{*}({Ɣ}^{*}+1)}{64\{\frac{1}{{ƛ}_{400}^{*}}+ʎ({Ɣ}^{*}+1)\}}-Ɱ\right)>0$$

## 6. Data description

In this part, we carry out a mathematical study to see the performances of proposed estimator in comparison of existing estimator. We considered two real data sets under SRS. The data descriptions of these populations are given in Table 1. We compare the production of our proposed estimator with existing counterparts in terms of PRE. To obtain the PRE, we used the following expression.


$$PRE(.)=\frac{Var({\hat{\bar{Y}}}_{0})}{MSE({\hat{\bar{Y}}}_{u})}\times 100$$

Where (u) = (0, 1, 2, 3, 4, 5, 6).

**Population 1:** [**Source:** Singh [41]]

*Y*: Estimated number of fish in year 1995.

*X*: Estimated number of fish in year 1994,

*R*_*x*_ = Rank of *X* variable

**Population 2:** [**Source:** Singh [41]]

*Y*: Estimated number of fish in year 1995.

*X*: Estimated number of fish in year 1993,

*R*_*x*_ = Rank of *X* variable

**Table 1:** Summary statistic of Populations 1–2.

	Populations	
Parameters	1	2
*N*	69	69
*n*	15	15
ʎ	0.0521739	0.0521739
$\bar{Y}$	4514.899	4514.899
$\bar{X}$	4954.435	4591.072
${\bar{R}}_{x}$	35	35
$S_{y}^{2}$	37199578	37199578
$S_{x}^{2}$	49829270	39881874
$S_{rx}^{2}$	402.5	402.5
*C*_*y*_	1.350893	1.350893
*C*_*x*_	1.424781	1.375541
*C*_*rx*_	0.5732115	0.5732115
*ρ*_*yx*_	0.9601401	0.9564207
*ρ*_*yrx*_	0.7688589	0.7539346
*ρ*_*xrx*_	0.7543462	0.7593837
${ƛ}_{400}^{*}$	6.544991	6.544991
${ƛ}_{040}^{*}$	8.69773	8.698631
${ƛ}_{004}^{*}$	0.747145	0.747145
${ƛ}_{220}^{*}$	7.053057	7.190264
${ƛ}_{202}^{*}$	1.159139	1.157465
${ƛ}_{022}^{*}$	1.298094	1.303701

## 7. Simulation study

We have generated two populations of size 5,000 from normal distribution with different parameters by using R language. The populations have different correlations, i.e. population I is positively correlated and population-II has strong positive correlation between *X* and *Y* variables. The population details are given below:

### Population-1

*X* = *N*(5,10), *Y* = *X*+*N*(2,8), *N* = 5,000, $\bar{Y}$ = 6.989187, $\bar{X}$ = 5.146998, ${\bar{R}}_{x}$ = 2500.5, $S_{y}^{2}$ = 156.7091, $S_{x}^{2}$ = 95.68973, $S_{rx}^{2}$ = 2083750, *ρ*_*yx*_ = 0.7696238, *ρ*_*yrx*_ = 0.7529799, *ρ*_*xrx*_ = 0.975884.

### Population-2

*X* = *N*(5,10), *Y* = *X*+*N*(1,3), *N* = 5,000, $\bar{Y}$ = 6.114148, $\bar{X}$ = 5.03637, ${\bar{R}}_{x}$ = 2500.5, $S_{y}^{2}$ = 104.8296, $S_{x}^{2}$ = 95.96678, $S_{rx}^{2}$ = 2083750, *ρ*_*yx*_ = 0.9551769, *ρ*_*yrx*_ = 0.9335775, *ρ*_*xrx*_ = 0.9764096.

The percentage relative efficiency (PRE) is calculated as follows:

$\mathrm{PRE}=\frac{Var({\hat{\bar{Y}}}_{0})}{MSE({\hat{\bar{Y}}}_{j})}*100$, where j = 0,1,…,6.

The results are given below:

## 8. Discussion

To show the performances of our suggested improved variance estimator, we used two real populations. Data description of these mentioned populations are given in Table 1. The bias result using real data sets are given in Table 2. The MSE and PRE are presented in Table 3. From the numerical results, the proposed estimator is found to be more effective than its existing estimators. The gain in efficiency in population 2 is more as compared to population 1. As we increase the sample size, the MSE decreases and PRE increases, yielding the expected results. We also evaluated the efficiency of our proposed improved variance estimator in simple random sampling using simulation study. The bias result using simulated data sets are given in Table 4. From Table 5, it is concluded that our suggested improved variance estimator performs better than the standard estimator and other existing estimators for a number of correlation coefficient values. The outcome of the simulation study clearly determines that for the simulated data sets 1–2, the PRE of the proposed estimator is greater than the PRE of the existing estimate. Overall the gain in efficiency of our proposed estimator is the best as compared to all existing counterparts.

**Table 2:** Bias result using Populations 1–2 using two real data sets.

Estimators	Population-I	Population-II
${\hat{\bar{Y}}}_{0}$	-	-
${\hat{\bar{Y}}}_{1}$	1.187433e+14	1.089019e+14
${\hat{\bar{Y}}}_{2}$	-	-
${\hat{\bar{Y}}}_{3}$	1.655189e+18	1.205829e+13
${\hat{\bar{Y}}}_{4}$	-1.912397e+13	-2.405279e+13
${\hat{\bar{Y}}}_{5}$	6.540944e+16	5.91082e+16
${\hat{\bar{Y}}}_{6}$	-1.269339e+46	-5.184275e+45
${\hat{\bar{Y}}}_{prop}$	5.168886e+16	5.564613e+16

**Table 3:** MSE and Percentage relative efficiency using Populations 1–2.

	Population-I		Population-II	
Estimators	MSE	PRE	MSE	PRE
${\hat{\bar{Y}}}_{0}$	4.725399e+14	100	4.725399e+14	100
${\hat{\bar{Y}}}_{1}$	8.206155e+13	575.8359	6.231377e+13	758.3234
${\hat{\bar{Y}}}_{2}$	5.960812e+13	792.7441	4.342992e+13	1088.052
${\hat{\bar{Y}}}_{3}$	5.714651e+14	826.8919	4.210837e+14	1122.199
${\hat{\bar{Y}}}_{4}$	1.203095e+13	392.7702	1.104193e+14	427.9503
${\hat{\bar{Y}}}_{5}$	4.639459e+13	1018.524	3.301273e+13	1431.387
${\hat{\bar{Y}}}_{6}$	4.518437e+13	1045.804	3.231554e+13	1462.268
${\hat{\bar{Y}}}_{prop}$	2.928899e+13	1613.37	2.600185e+13	1817.332

**Table 4:** Bias result using Populations 1–2 using simulated data sets.

Estimators	Population-I	Population-II
${\hat{\bar{Y}}}_{0}$	-	-
${\hat{\bar{Y}}}_{1}$	199.1479	9.756393
${\hat{\bar{Y}}}_{2}$	-	-
${\hat{\bar{Y}}}_{3}$	12722895	1619653
${\hat{\bar{Y}}}_{4}$	39.87267	-21.2332
${\hat{\bar{Y}}}_{5}$	465181.2	136970.7
${\hat{\bar{Y}}}_{6}$	-1.156381e+19	-3.046733e+20
${\hat{\bar{Y}}}_{prop}$	-128030.3	-5891.692

**Table 5:** MSE and PRE of ${\hat{\bar{Y}}}_{prop}$, w.r.t the existing counterparts using two simulated data.

	Population-I		Population-II	
Estimators	MSE	PRE	MSE	PRE
${\hat{\bar{Y}}}_{0}$	185.8621	100	172.0718	100
${\hat{\bar{Y}}}_{1}$	135.8605	136.8036	30.20478	569.6842
${\hat{\bar{Y}}}_{2}$	109.091	170.3734	28.44441	604.9408
${\hat{\bar{Y}}}_{3}$	107.9588	172.1601	28.36553	606.6230
${\hat{\bar{Y}}}_{4}$	112.3095	165.491	56.84092	302.7253
${\hat{\bar{Y}}}_{5}$	107.3989	173.0577	28.19487	610.2948
${\hat{\bar{Y}}}_{6}$	107.3748	173.0966	28.18003	610.6162
${\hat{\bar{Y}}}_{prop}$	70.44824	263.8278	24.17502	711.7754

## 9. Conclusion

In this article, we proposed an improved ratio-in-regression type exponential estimator for the finite population variance using dual auxiliary variables under simple random sampling. Expressions for mean square error of the proposed estimator are derived up to first order of approximation. The proposed estimator is compared with several existing counterparts to judge their uniqueness and superiority using two real data sets. Moreover, simulation study is also conducted to check the robustness and generalizability of the proposed variance estimator. A numerical study is carried out to support the theoretical results. Based on the numerical findings, it is observed from the result of real data sets and simulation study, showing when compared to its existing counterparts; the suggested estimator produces well results. Therefore we recommend the use of our proposed estimators for efficiently estimating the finite population varaince under simple random sampling using dual auxiliary varaible. The current work can be extended to develop an improved class of estimators under measurement error, probability proportional to size (PPS), two-stage sampling using dual auxiliary variable for estimating the population variance.
